# Socio-demographic determinants of the knowledge of Monkeypox Virus among the general public: a cross-sectional study in a Tertiary Care Center in Nepal

**DOI:** 10.1186/s12879-024-09184-5

**Published:** 2024-03-06

**Authors:** Santa Kumar Das, Abhinav Bhattarai, Kiran Paudel, Sandesh Bhusal, Sangam Shah, Sakchhyam Timsina, Aastha Subedi, Sandhya Niroula, Najim Z. Alshahrani, Sanjit Sah, Bijaya Kumar Padhi, Joshuan J. Barboza, Alfonso J. Rodriguez-Morales, Luis A. Salas-Matta, D. Katterine Bonilla-Aldana, Ranjit Sah

**Affiliations:** 1 Department of Pulmonology and Critical Care, Tribhuvan University, Institute of Medicine, 44600 Maharajgunj, Nepal; 2 Institute of Medicine, Tribhuvan University, Kathmandu, Nepal; 3 Central Department of Public Health, Institute of Medicine, Tribhuvan University, Kathmandu, Nepal; 4 Nepal Health Frontiers, Tokha-5, Kathmandu, Nepal; 5 Department of Family and Community Medicine, Faculty of Medicine, University of Jeddah, 21589 Jeddah, Saudi Arabia; 6 Research Scientist, Global Consortium for Public Health and Research, Datta Meghe Institute of Higher Education and Research, Jawaharlal Nehru Medical College, 442001 Wardha, India; 7 SR Sanjeevani Hospital, Kalyanpur-10, Siraha, Nepal; 8 Department of Community Medicine and School of Public Health, Postgraduate Institute of Medical Education and Research (PGIMER), Chandigarh, India; 9 School of Medicine, Universidad Norbert Wiener, Lima, Peru; 10 Faculties of Health Sciences and Environmental Sciences, Universidad Científica del Sur, 4861 Lima, Peru; 11 Gilbert and Rose-Marie Chagoury School of Medicine, Lebanese American University, Beirut, P.O. Box 36, Lebanon; 12 Grupo de Investigación Biomedicina, Faculty of Msedicine, Fundacion Universitaria Autónoma de las Américas-Institucion Universitaria Vision de las Americas, Pereira, Risaralda Colombia; 13 Research Unit, Universidad Continental, Huancayo, Peru; 14 Department of Microbiology, Tribhuvan University Teaching Hospital, Institute of Medicine, 44600 Kathmandu, Nepal; 15 Department of Microbiology, Dr. D. Y. Patil Medical College, Hospital and Research Centre, Dr. D. Y. Patil Vidyapeeth, 411018 Pune, Maharashtra India; 16 Department of Public Health Dentistry, Dr. D.Y. Patil Dental College and Hospital, Dr. D.Y. Patil Vidyapeeth, 411018 Pune, Maharashtra India

**Keywords:** Knowledge, Monkey pox, Nepal, Virus, MPXV, Population

## Abstract

**Background and objective:**

Monkeypox virus (MPXV) is the causative agent of monkeypox’s zoonotic infection and was declared a global emergency by the World Health Organization (WHO). Studies from different countries have shown insufficient knowledge among the general public on MPXV. This study aimed to assess the knowledge of the general public of Nepal on MPXV.

**Methods:**

Three hundred people were interviewed in person in October 2022, and 282 complete responses were recorded. The questionnaire related to the knowledge of MPXV was derived from a previous study conducted among the general population of Saudi Arabia. Twenty-two questions were included that assessed the knowledge and attitude of Nepalese toward monkeypox. Statistical comparison between high and low knowledge was performed using Pearson’s Chi-square test. Logistic regression models were deployed to establish the relationship between participants’ knowledge and socio-demographic characteristics.

**Results:**

Among the total respondents, 53.8% demonstrated high knowledge of monkeypox. People aged 18–25 years, unmarried people, and those living in urban areas had significantly higher levels of knowledge. Most respondents believed that MPXV is not a conspiracy or bioterrorism (63.1%) and agreed that it is likely to affect people’s social and economic life as COVID-19 did (67.0%). The history of COVID-19 vaccination (aOR: 2.980; 95%CI: 1.227, 7.236) and the younger age (aOR: 2.975; 95%CI: 1.097, 8.069) were found to be significant determinants of the knowledge of the participants on monkeypox.

**Conclusion:**

We observed that most Nepalese populations had a high knowledge of monkeypox and that social media was the most valuable source of information.

## Introduction

Monkeypox virus (MPXV) is a double-stranded DNA virus that belongs to the Poxviridae family and causes a zoonotic infection. MPXV belongs to the same family as smallpox, and symptoms of both resemble clinically [1]. The World Health Organization (WHO) declared monkeypox (MPXV) a global health emergency, considering monkeypox outbreaks in more than 100 countries [2]. Globally, as of February 2024, the total number of MPXV cases reported from 118 countries has exceeded 93,000. The highest number of cases has been reported from the United States, with over 31,000 confirmed cases. One hundred eleven countries where cases have been reported do not have a history of previous MPXV outbreaks and are experiencing their first outbreak. Seven have already had a history of sporadic outbreaks [3]. On November 28, 2022, WHO put forward the term ‘mpox’ as the preferred terminology for monkeypox [4].

Today, media coverage has reported MPXV as a novel virus, which is a false claim. It was discovered for the first time in 1958 in a colony of Asian monkeys kept for research in an animal facility at the Statens Serum Institute, Copenhagen, Denmark [5]. In 1970, the first human infection with MPXV was reported in a 9-month-old boy in the Democratic Republic of Congo (then Zaire), where smallpox had already been eradicated. Subsequently, sporadic outbreaks have occurred in Central and West Africa [6]. The first monkeypox outbreak outside the African continent occurred in 2003 in the USA, which was attributed to the importation of infected pets [7]. However, the largest outbreak occurred in Nigeria in 2017, with 68 confirmed cases. A Nigerian tourist reported Asia’s first monkeypox infection in Singapore in 2019 [8]. Similarly, multiple cases of monkeypox have been reported.

The new, unanticipated rise in monkeypox cases began in early May 2022, with a sharp increase in cases in Europe, the United States, Australia, and most other non-endemic countries, causing a global public health concern. Despite the WHO’s declaration of a public health emergency on July 23, 2022, and the continuous implementation of preventive measures to oversee the outbreak, cases have not subsided and rapidly increased until 2023. Until early August 2022, 30,000 cases were confirmed; within two months, by October, the cases doubled [3]. On the mortality side, the death rate is lower than expected, representing 0.04% [9], significantly lower than the 1–3% reported during outbreaks caused by a similar viral strain in West Africa over the past few decades [9].

Although the typical route of infection with MPXV is zoonotic, easy person-to-person transmission has been shown. Direct contact with MPXV infected person, including rashes, scabs, or body fluids, can result in the transmission of the disease. Furthermore, contact with inanimate objects, such as contaminated fabrics, linens or towels, can cause an infection. Sexual contact and intimate social behaviours, including hugging, kissing, and handshakes, could be sources of direct contact [10, 11]. Transmission via respiratory secretions has been documented. However, the case-fatality rate is much lower than smallpox (MPXV 0-11%, while smallpox is up to 30%) [12, 13]. Common symptoms of MPXV are fever, body pain, headache, rashes on the skin, and lymphadenopathy. In particular, the rashes typically on the skin, scalp, and genitals come to visibility, after which MPXV infection is suspected. However, life-threatening complications of MPXV, such as pneumonia, secondary skin infections, proctitis, ocular problems, and sepsis due to bacterial superinfections, may occur [14]. Currently, the definitive treatment for MPXV is unavailable; antiviral drugs used for smallpox treatment and smallpox vaccines are used to treat MPXV.

Although MPXV is not a novel virus, its outbreaks have been occurring for a long time. Until now, there have been no cases confirmed in Nepal yet. However, the rapid incline in cases in neighbouring India is concerning [3]. Since the Nepal-India border is open, Nepal appears to be at a high risk of transmission and outbreak. Nepal’s progress on MPXV preparedness is continuous. The National Public Health Laboratory (NPHL), Kathmandu, has upgraded its diagnostic tools and protocols to comply with WHO MPXV detection standards to avoid missing any cases.

Furthermore, the Ministry of Health and Population, Nepal, has established a hotline for reporting febrile and pox-like symptoms doubtful of MPXV infection [15]. Importantly, Nepal is prone to suffering from unhealable health and economic crisis in case of an outbreak, as observed by the COVID-19 lockdowns. Above all the preparedness of health authorities against MPXV, there has to be sufficient knowledge and comprehension of every citizen on disease outbreaks, currently as MPXV. Unfortunately, a decline in research and awareness has resulted in illiteracy and negligence of the potentially contagious infection.

During the pandemic of COVID-19, MPXV could emerge as an additional burden to the world in terms of health and economy. In addition, the cessation of smallpox vaccination has left a significant portion of the global population susceptible to MPXV, with increasing cases in Central and West African countries [16]. Nepal’s vulnerability to MPXV is a concern due to its lack of smallpox vaccination program. People need to have adequate knowledge of this emerging MPXV outbreak to achieve the objectives of reducing cases and preventing the spread of a new burden. From our COVID-19 experiences, inadequate awareness and increased public negligence were two major factors responsible for the rapid rise in cases despite strict preventive measures such as lockdowns and vaccination. Reports from WHO state that a challenge in MPXV outbreak control is citizens’ lack of sufficient knowledge [17]. In this regard, many studies investigated the level of knowledge about MPXV among various diminutions of people of different nations [18]. Most of these studies have found that most of the population has inadequate knowledge of MPXV [11, 12, 19, 20]. Currently, there are no studies investigating the knowledge of Nepalese citizens on monkeypox. Therefore, our primary objective was to assess the level of knowledge about MPXV among the general population of Nepal and establish its association with the population’s socio-demographics. Additionally, we investigated the difference in the knowledge according to different sources of information.

## Methods

### Study design

A cross-sectional single-centred study was conducted among people who visited Tribhuvan University Teaching Hospital (TUTH) in Maharajgunj, Kathmandu, Nepal, in October 2022. TUTH is one of the largest health institutions in Nepal and is known to provide a wide variety of disciplines and medical services for which people from all around Nepal arrive for consultation and treatment. The selection of this study site increased our chances of inclusion of a diverse Nepalese population. Moreover, since all investigators had been affiliated with the study site, this site was selected for easy data acquisition. The research was performed in a qualitative cross-sectional study design.

### Ethical approval and consent to participate

The Institutional Review Board (IRB) of the Institute of Medicine (IOM) has reviewed, revised, and provided the final ethical approval for the study (Approval number: 183 [6–11]E2). All methods were carried out in accordance with declaration of Helsinki guidelines and regulations. Written informed consent was obtained from the participants for the data collection.

### Study participants and eligibility criteria

**Fig. 1:**
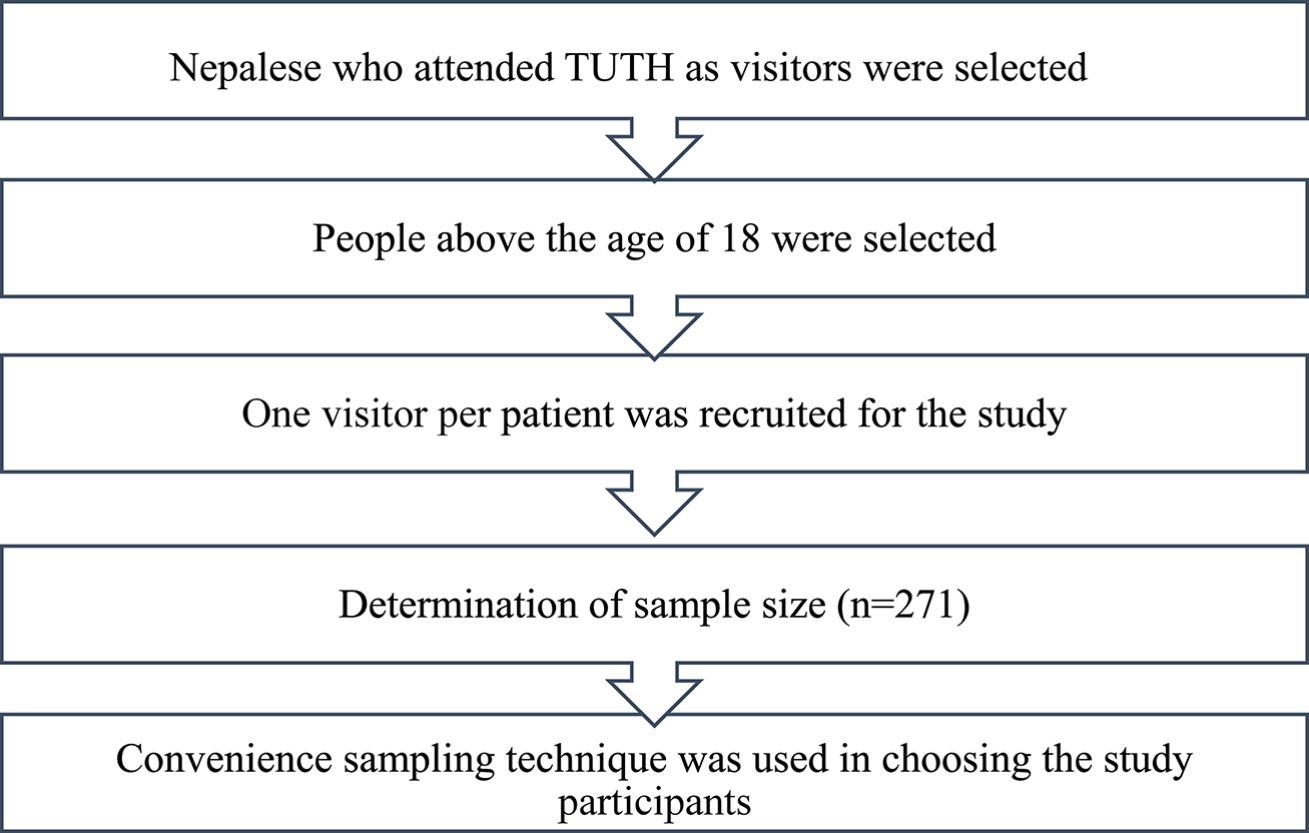
Selection of study participants

All Nepalese over 18 years who attended TUTH as visitors were the desired inclusive population, and therefore, a non-probability convenience sampling strategy was utilised. Visitors at the hospital’s visitor section were interviewed, and one visitor per patient was recruited for the study. Those who were willing to give their consent were eligible to be included in this study. Likewise, sick individuals who visited the hospital for consultation were also excluded. (Fig. 1) We used a convenience sampling technique in choosing the study participants. The sample size was calculated using the formula;

$${\rm{n}}\,{\rm{ = }}\,{{\rm{Z}}^{\rm{2}}}{\rm{p}}\,\left( {{\rm{1}}\, - \,{\rm{p}}} \right)\,{\rm{/}}\,{{\rm{d}}^{\rm{2}}}$$

z = standard normal variate is 1.96 at a 95% confidence level.

p = expected proportion in population based on previous study or pilot study.

d = desirable error = 5%.

To our knowledge, this is the first study conducted in Nepal. Thus, considering the conservative estimate of 20% with a precision error of 5% and a 95% confidence level.

$$\eqalign{{\rm{Sample}}\,{\rm{size,}}\,{\rm{n}}\,{\rm{ = }} & \,{\left( {{\rm{1}}{\rm{.96}}} \right)^{\rm{2}}}\,{\rm{ \times }}\,{\rm{0}}{\rm{.2}}\,{\rm{ \times }}\,\left( {{\rm{1}}\, - \,{\rm{0}}{\rm{.2}}} \right)\,{\rm{/}}\,{\left( {{\rm{0}}{\rm{.05}}} \right)^2} \cr & = \,246 \cr} $$

Considering the non-response rate as 10%, the non-response rate = 10% of 246 = 24.6 25.

Therefore, the minimum sample size = 246 + 25 = 271.

### Data collection tool

We used a structured, validated questionnaire for data collection. The questions referenced a previously published study conducted by Alshahrani et al. in Saudi Arabia [19]. We modified it to make it suitable in the context of Nepal. For the ease of study participants, the questions were translated into the Nepali language. Initially, authors KP, SS, and AS, who held extensive experience in community interviews, tested the quality and reliability of this survey. Investigators ST, AS, and SB conducted one-to-one interviews to complete the questionnaire. The questionnaire had two sections: the first covered socio-demographic details and health-related questions. Twenty-two multiple-choice questions measuring knowledge of monkeypox were included in the second portion. They were developed based on prior research [16, 18, 20–24] and existing facts from the United States Centers for Disease Control and Prevention (CDC) [25]. Each question included three options for responses: “Yes,” “No,” and “I don’t know.” To ensure the validity of this survey, it was pre-tested on 15 members of the general public. The pilot study results were only used to enhance the clarity of the questions.

### Data collection procedure

Information from the general public was gathered via a standardised questionnaire. The investigators and volunteers collected data from the respondents *via* an in-person survey. Before proceeding to the interview, each respondent was asked to consent by signing the consent statement. The objectives and the expected benefits of the study were clearly explained. After the acquisition of consent, an in-person survey was performed, where each question was asked to the respondent, and answers were noted. Each in-person survey took approximately 8–10 min.

### Study variables

Twenty-two questions with “Yes,” “No”, and “I don’t know” responses were used to assess the knowledge of monkeypox. Scores were assigned as ‘1’ for correct answers and ‘0’ for incorrect answers. If the participant selected ‘I don’t know’, the selection was assigned string ‘NA’ and the particular question was excluded from the knowledge score evaluation. The knowledge score was classified as 0 (lowest) to 22 (highest).

Age, which was divided into categories of 18 to 25 years, 26 to 45 years, and over 46 years, gender (male or female), marital status (single or married), region of residence region in Nepal (central / mid-western / far-western / western/eastern) and urban or rural area, were explanatory variables. The employment status, the nature of their job as healthcare workers, the presence of chronic conditions, their smoking habits, and their monthly salary were all asked about. The amount of income was calculated in Nepalese rupees (NR) and split into two ranges: equal or more than 30,000 (≥ US$227) and less than 30,000 (< US$227). The levels of education were recorded as high school and lower, bachelor’s and postgraduate degrees. They were asked if they had received every recommended childhood vaccination. The participants had to select all relevant sources from TV and radio, social media, healthcare professionals, family or friends, books, studies, and articles to determine where they learned about monkeypox. Following questions about monkeypox symptoms, participants were asked if they believed the disease would have the same social and economic effects as the COVID-19 pandemic or if it was a plot or act of bioterrorism.

### Statistical analysis

We used IBM SPSS Statistics, Version 26 (IBM Corp, Armonk, NY, USA) for statistical analyses. Mean and standard deviation (SD) was used to represent continuous variables, and frequency and percentages were used to represent categorical variables. The data, specifically the knowledge scores, followed a normal distribution with mean and SD. Furthermore, knowledge scores were divided into low and high levels of knowledge. As a cut-off point, we used the mean score of 10; a mean score > 10 was considered high, and a mean score of 10 or less was considered low. Pearson’s Chi-square test was performed to compare the explanatory and response variables. Multivariable analysis was performed to analyse the association of different variables on the knowledge of MPXV. Since there are always possibilities of suppressor variables that could suppress the statistical significance in univariable analysis, all variables were subjected to the multivariable analysis. The threshold for statistical significance was established using the p-value < 0.05.

## Results

### Socio-demographic characteristics of the study participants

Of the 300 questionnaires distributed, 282(94%) were completed and included in the final analysis. Most respondents (50.4%) were 18–25 years old. Male respondents were greater than females (55.7% and 44.3%, respectively), and most were unmarried (56.4%). Most of the respondents (67.4%) resided in urban areas of Nepal. A large proportion (69.1%) of the respondents were not graduates, followed by postgraduates (25.9%) and bachelor’s degrees (5.0%). Most were unemployed (63.8%), and most employed had a monthly income of less than 30 thousand Nepalese rupees (69.1%).

Among the participants, 17.4% had an existing chronic disease, and 18.1% were smokers. More than 90% of all respondents had completed all childhood vaccinations provided by the Nepalese government, including the recent COVID-19 vaccination. Most respondents believed that MPXV is not a conspiracy or bioterrorism (63.1%) and agreed that it is likely to affect people’s social and economic life as COVID-19 did (67.0%) (Table 1).

**Table 1:** Relationship between the knowledge score and the socio-demographics of the respondents

Variable	N (%)	Knowledge score	Knowledge level (cut-off score = 10)		
		Mean (SD)	Low (*n* = 132)	High (*n* = 150)	P-value
Age in Years					
18–25	142 (50.4)	11.6 (3.5)	53 (37.3)	89 (62.7)	0.003*
26–45	111 (39.4)	10.0 (4.3)	60 (54.1)	51 (45.9)	
45 above	29 (10.3)	8.5 (4.6)	19 (65.5)	10 (34.5)	
**Gender**					
Male	157 (55.7)	10.8 (4.1)	74 (47.1)	83 (52.9)	0.9
Female	125 (44.3)	10.5 (4.2)	58 (46.4)	67 (53.6)	
**Marital Status**					
Married	123 (43.6)	9.7 94.4)	68 (55.3)	55 (44.7)	0.01*
Unmarried	159 (56.4)	11.4 (3.7)	64 (40.3)	9 (59.7)	
**Residency**					
Urban	190 (67.4)	11.2 (3.8)	80 (42.1)	110 (57.9)	0.02*
Rural	92 (32.6)	9.7 (4.4)	52 (56.5)	40 (43.5)	
**Education level**					
High school and below	195 (69.1)	10.5 (4.1)	93 (47.7)	102 (52.3)	0.9
Undergraduate	14 (5.0)	11.1 (4.0)	6 (42.9)	8 (57.1)	
Postgraduate	73 (25.9)	10.9 (4.5)	33 (45.2)	40 (54.8)	
**Employment**					
Yes	102 (36.2)	9.5 (3.6)	48 (47.1)	54 (52.9)	0.9
No	180 (63.8)	10.5 (5.4)	84 (46.7)	96 (53.3)	
**Monthly Income**					
Below 30 thousand	195 (69.1)	11.6 (3.1)	91 (46.7)	104 (53.3)	0.9
30 thousand and above	87 (30.9)	10.8 (2.6)	41 (47.1)	46 (52.99	
**Chronic disease**					
Yes	49 (17.4)	9.8 (5.4)	26 (53.1)	23 (46.9)	0.3
No	233 (82.6)	10.8 (3.8)	106 (45.5)	127 (54.5)	
**Health care worker**					
Yes	16 (5.7)	11.3 (3.7)	7 (43.8)	9 (56.2)	0.8
No	266 (94.3)	10.6 (4.1)	125 (47.0)	141 (53)	
**Do you ever smoke?**					
Yes	51 (18.1)	10.0 (3.7)	28 (54.9)	23 (45.1)	0.2
No	231 (81.9)	10.8 (4.1)	104 (45.0)	127 (55.0)	
**COVID-19 vaccinated**					
Yes	255 (90.4)	10.9 (4.0)	113 (44.3)	142 (55.7)	0.01*
No	27 (9.6)	8.3 (4.6)	19 (70.4)	8 (29.6)	
**Childhood vaccinated**					
Yes	258 (91.5)	10.8 (4.1)	115 (44.6)	143 (55.4)	0.01*
No	24 (8.5)	8.8 (3.9)	17 (70.8)	7 (29.2)	

*Significant p value < 0.05

### Relationship between the knowledge score and the socio-demographics of the respondents

The proportion of respondents and the answers recorded is displayed in Table 2. The level of knowledge was classified as low and high based on the cut-off value, which was 10 in our study. One hundred thirty-two individuals (46.8%) and 150 individuals (53.2%) had low and high knowledge, respectively. Age (*p* = 0.003), marital status (*p* = 0.01), residency area (*p* = 0.02) and vaccination status (*p* = 0.01) were significantly associated with the difference in knowledge of monkeypox. Unmarried respondents aged 18–25, respondents living in urban areas, and respondents with complete childhood and COVID-19 vaccination had a significantly higher level of knowledge (*p* < 0.05). Knowledge did not differ between male and female respondents (*p* = 0.9). There were no significant differences in monkeypox knowledge in terms of educational qualifications. The majority of nonsmokers had a higher knowledge score (55.0%). Most respondents (53.8%) who agreed that monkeypox is a conspiracy or bioterrorism had a lower level of knowledge (Table 1).

**Table 2:** Responses for knowledge questions

S.N	Question	Yes (%)	No (%)
1	Is monkeypox an infectious disease?	89.5	10.5
2	Monkeypox is a new infection that appeared in the year 2022	19.1	80.9
3	Monkeypox is a sexually transmitted disease	48.6	51.4
4	Chickenpox and monkeypox are the same diseases.	62.1	37.9
5	Monkeypox is common in Middle Eastern countries.	29.4	70.6
6	Monkeypox is common in West and Central African countries.	49.6	50.4
7	There are many cases recorded in Nepal.	55	45
8	Monkeypox cases are increasing in the USA and Europe	51	49
9	Monkeypox is a contagious viral disease	69	31
10	Monkeypox is a contagious bacterial disease	44.7	55.3
11	Monkeypox is easily transmitted from one person to another.	12.1	87.9
12	Monkeypox is transmitted to humans through bites and scratches from infected animals.	53	47
13	People with monkeypox can transmit the disease to others (the disease is transmitted between humans).	67.4	32.6
14	Monkeypox is spread by droplets (coughing and sneezing)	48	52
15	The first symptoms of monkeypox are similar to the flu	47	53
16	Monkeypox only affects males	73	27
17	Hand sanitisers and face masks are important in preventing monkeypox	64	36
18	There is a special treatment for monkeypox	29	71
19	Monkeypox is spread through bodily fluids	49	51
20	There is a monkeypox vaccine available in Nepal	54	45
21	The chickenpox vaccine I got in childhood protects me from monkeypox.	46	54
22	There is a smallpox vaccine that can be used for monkeypox.	17	82

### Relationship between the knowledge score and the source of information of the participants

Respondents who learned about the MPXV and its outbreak via social media had significantly higher knowledge than those who knew about MPXV via television and radio or other individuals, including healthcare providers and family or friends (Table 3).

**Table 3:** Relationship between the knowledge score and the source of information of the participants

Source of information	Knowledge level		P value
**TV and radio**	Low	High	
Yes	94 (61.4)	59 (38.6)	< 0.001*
No	38 (29.5)	91 (70.5)	
**Social Media**			
Yes	94 (42.5)	127 (57.5)	0.006*
No	38 (62.3)	23 (37.7)	
**Health care provider**			
Yes	34 (68%)	16 [32]	0.001*
No	98 (42.2)	134 (57.8)	
**Family or friend**			
Yes	24 (48)	26 (52)	0.8
No	108 (46.6)	124 (53.4)	

Table 4 shows the multiple logistic regression analysis. The history of COVID vaccination (aOR: 2.980; 95%CI: 1.227, 7.236) and younger age (aOR: 2.975; 95%CI: 1.097, 8.069) were found to be significant determinants of the knowledge of the participants on monkeypox.

**Table 4:** Multiple logistic regression analysis for association between knowledge and socio-demographic characteristics (*N* = 282)

Variable	aOR [95% CI]	P-value
**Age**		
18–25	**Ref**	
26–45	2.975 [1.097, 8.069]	0.032*
45+	1.401 [0.582, 3.372]	0.451
**Gender**		
Male	**Ref**	
Female	1.035 [0.630, 1.699]	0.892
**Education**		
PG	**Ref**	
UG	1.083 [0.328, 3.570]	0.896
High school and below	0.907 [0.517, 1.591]	0.733
**Employment**		
No	**Ref**	0.6
Yes	1.1 (0.6–2.1)	
**Income**		0.896
<30,000 (NRs)	**Ref**	
≥30,000 (NRs)	1.374 [0.766, 2.462]	0.286
**Marital Status**		
Married	**Ref**	
Unmarried	1.175 [0.608, 2.270]	0.630
**Residence**		
Rural	**Ref**	0.7
Urban	1.1 [0.5, 2.0]	
**Healthcare worker**		
No	**Ref**	
Yes	1.5 [0.7, 2.9]	0.20
**Chronic disease**		
No	**Ref**	
Yes	1.4 (0.6–3.1)	0.37
**Smoking**		
No	**Ref**	
Yes	0.8 (0.4–1.8)	0.7
**History of COVID vaccination**		
No	**Ref**	
Yes	2.980 [1.227, 7.236]	0.015*
**History of childhood vaccination**		
No	**Ref**	
Yes	2.6 [0.9, 7.7]	0.07

## Discussion

Our findings suggest that the general public lacks critical knowledge and comprehension of monkeypox. More than 53.2% of the study participants knew what monkeypox was, whereas 46.8% had never heard of the virus before. The general lack of information is unsurprising, given that monkeypox is a recurrent infectious disease and that no cases have ever been reported in Nepal. Our study aligns with prior researches in Nepal, indicating a inadequacy of information regarding monkeypox [26, 27]. Our findings differ from those of the Saudi population, which revealed that only 48% of respondents had a high knowledge of MNXP [19]; this disparity may be due to differences in study timing. In addition, our findings contrast with those of a Malaysian study, which found that despite their lack of knowledge of transmission and treatment, nearly 95% of respondents were aware of dengue fever, another viral infection, during its outbreak [28]. A pilot study carried out among hospital visitors in Karachi, Pakistan, showed that only 38.5% of participants understood the viral disease well. However, 90% of them were aware of it [29]. Similarly, a Pakistani poll found that only 35% of people in low and high-socioeconomic groups were aware of the dengue outbreak [30]. For the sake of public health, more research is required to understand why the endemic population around the world is so ignorant of a newly emerging virus [31].

Amid this global upheaval, the word about possible human monkeypox virus infections in Nepal spread quickly on social media. However, Nepali authorities denied these claims, claiming that no cases of monkeypox had been discovered yet. Given that the virus is still being found and people are still dying of it, it is urgent to learn more about its source and how it spreads and to provide people with the information and help they need to protect themselves and others in various situations [32]. So far as we know, this is the first survey ever done in Nepal to determine how much the general public knows about the basics, spread, transmission, symptoms, prevention, and treatment of human monkeypox virus infection.

Additionally, most of our study participants were largely unaware of the history of the virus, and there is typically not enough comprehension of the transmission channels. Our results corroborate the World Health Organization (WHO) study that found that one of the challenges in preventing the re-emergence of monkeypox was a lack of knowledge about the disease [33]. Although most survey respondents could not distinguish between monkeypox and smallpox symptoms, practically all of them were aware that monkeypox is a viral disease that affects them. However, due to a lack of information, the Nepali population is primarily unaware of the types and diversity of viruses that are widespread worldwide.

Our results also showed that people who rely on the internet or social media for information are more knowledgeable about monkeypox than their peers. That might result from the ease with which most people can access information that has been updated thanks to social media and the internet. That demonstrates the internet’s value in promoting health, particularly in pandemic situations [34]. Online media have become one of the most essential and convenient means of accessing information compared to other options [35]. These results are consistent with those of further research carried out in Ethiopia [36] and Egypt [37], which identified the internet and social media as the primary sources of information. Compared to other media, newspapers, local authorities, and healthcare professionals appear to be less prevalent sources of information about the disease. As a result, to effectively convey knowledge to the public, the Nepali health system may strengthen the participation of community leaders and healthcare workers.

Keeping with the bulk of earlier study conducted in the underdeveloped countries [38, 39], we found that men in our study were more knowledgeable about this viral infection than women. As reported in earlier studies [40–42], we found that participants’ knowledge of the transmission of this virus increased among participants along with their degree of education. The finding of one Chinese study [43] is consistent with this finding. That may be explained by greater accessibility to media such as television, radio, and online social networks [44]. The availability of appropriate facilities to support health educators, including medical experts and government officials, may also impact the general public’s level of understanding in this sector.

Since Nepal is a multi-ethnic country with substantially divergent economic circumstances, educational attainment levels, and cultural norms, it is expected that the population’s levels of knowledge would also differ considerably [45]. Population segments without internet access or who live in places where the fast escalation of transmission is less likely will also demonstrate less knowledge when standard and uniform education and dissemination measures are advocated for and put into practice. That is accurate despite a sizeable section of the sample containing good knowledge. Our study holds some limitations. The study was conducted in just one tertiary care centre, which limits the representativeness of the results to other settings. Likewise, we had to deploy convenience sampling due to resource constraints and could not accommodate a larger sample size, which might limit a more comprehensive and generalisable result.

## Conclusions

Although mpox is no longer a Public Health Emergency of International Concern, it is still a differential diagnosis, and transmission occurs globally [44, 45]. Then, knowledge of this viral disease is necessary to correctly approach disease prevention, control and treatment among healthcare workers and the general population. Around 54% of the participants had high knowledge of the Monkeypox virus. Unmarried respondents aged 18–25 and those living in urban areas had significantly higher knowledge. Those vaccinated for COVID-19 and all childhood vaccines had comparatively good knowledge. Most people who had good knowledge received information on social media. Overall, the comprehension of the Nepalese population toward monkeypox is inadequate, and our findings reveal that there lies a crucial role of social media in uplifting the knowledge of the Nepalese population on monkeypox via extensive awareness and education so that public health safety amidst the monkeypox health emergency can be ensured. Furthermore, prospective studies in Nepal and the entire globe requires investigation into the improvement in the knowledge of the population regarding monkeypox over time.

This study lays the groundwork for forthcoming inquiries that can guide the development of focused educational initiatives, policy frameworks, and healthcare interventions. These efforts aim to enhance global preparedness and response capabilities in the face of emerging infectious diseases such as monkeypox. In essence, proactive measures are urgently needed to enhance public knowledge, preparedness, and response, fostering a more resilient and informed society. Future research should assess the long-term effectiveness of awareness campaigns in enhancing monkeypox knowledge in Nepal. Understanding vaccine acceptance and cultural factors influencing perceptions is crucial for targeted interventions. Comparative studies across diverse demographics and regions, exploration of integrating monkeypox information into school curricula, and deeper analysis of social media’s role in public understanding are recommended.

## Data Availability

The datasets used and/or analysed during the current study available from the corresponding author on reasonable request.
